# Hospital and Institutional News

**Published:** 1913-07-12

**Authors:** 


### HOSPITAL AND INSTITUTIONAL NEWS.

### THE KING AND QUEEN IN LANCASHIRE.

During the tour of the King and Queen in Lancashire in the early part of this week they received a touching and exhilarating welcome from the patients in the Southport Infirmary. The beds of several patients had been brought out on to the pavement, and cries of greeting and the waving of flags testified to a lively loyalty which sickness could not obscure. The procession of motor-cars slowed down as the infirmary was passed, and the Royal party graciously acknowledged the salutations. The voluntary system owes so much to Royalty, from the days when King Edward was Prince of Wales to the present time, that a hospital welcome to members of the Royal Family has always a particular flavour of intimacy, of which the above greeting is a happy and spontaneous instance.

### RETIREMENT OF A LIVERPOOL SECRETARY.

Mr. Joseph Unsworth has retired from the position of secretary and superintendent of the David Lewis Northern Hospital, Liverpool. Mr. Unsworth, who has held this post for the remarkable period of forty-five years, was presented at a meeting of the committee last week with a cheque which represented the joint donations of the members of the committee and other friends. The following resolution was passed unanimously: "That this committee desire to express their high appreciation of the long and faithful service of Mr. Joseph Unsworth, their secretary and superintendent, who for forty-five years has devoted his best energies to the service of the hospital, and in asking him to accept the enclosed cheque, trust he will have many years of rest and happiness." During his period of office the institution has experienced great changes and developments, the most important of which was, perhaps, its rebuilding in 1896-7. The number of beds in the institution at the present time is 200.

### BRITISH HOSPITAL FOR INCURABLES: NEW WING OPENED.

The Princess Royal opened last Monday the new Queen Alexandra wing at the British Home and Hospital for Incurables, Streatham. Unfortunately, rain interfered with the ceremonial arrangements, and the company had to take refuge in the recreation hall of the institution. Mr. Edwin Hall, F.R.I.B.A., the architect, handed the key to the Princess, and Mr. F. A. Bevan read an address, recalling that the new wing commemorates the jubilee of the institution, which, by the way, claims to be the first to gain the patronage of Queen Alexandra on her arrival in this country. The new wing provides accommodation for thirty-five patients, and was inspected by the Princess, with whom were the Duchess of Fife and Princess Maud. In the recreation room several purses were presented, one of which, offered by Mrs. Penman, contained two anonymous cheques for £1,000, which will endow two beds in the new wing. We condole with Mr. F. A. Bevan, the chairman, and with Mr. Edgar Penman, the secretary, on the bad weather, which robbed the ceremony of much picturesqueness, to secure which much trouble and pains had been taken. It would only be fitting, therefore, if all who were present made a special contribution to the institution's funds as a practical recognition of the efforts which the rain treated so unkindly.

### PAYING PATIENTS IN SOUTH AFRICA.

Our readers will note with interest a letter in our correspondence columns this week from Mr. G. S. Thompson, F.R.C.S., in which he takes up the question of paying patients and describes the way in which the system of paying patients is in force at Kimberley Hospital, South Africa. Now that Sir William Osler's address to the British Hospitals Association has attracted general attention to this subject, Mr. Thompson's practical details have an added interest and weight. We are particularly glad to publish his letter at the present time, and it is noteworthy that he emphasises, as Sir William Osler has done, the advantages which the hospital must have over the ordinary nursing home, and how the existence of a graduated scale provides, in conjunction with free patients, for all classes of the sick. He also draws attention to the help which the better class of paying patient gives towards the maintenance of the free patient. The system of graduated payments which prevails in several of the South African institutions is not so well known in this country as is the American system, and therefore this letter has not merely a timely but somewhat original interest in the present discussion.

### DEATH OF A BRISTOL HOSPITAL'S PRESIDENT.

The death of Mr. Joseph Storrs Fry, the head of the firm of cocoa manufacturers, which took place on Monday night, removes one of the most prominent supporters of the voluntary system in Bristol, a city which has always been a keen centre of hospital life and work. Mr. Fry, who was born in 1826, was the grandson of the founder of the business, and succeeded to the head of it in 1885. As a prominent Quaker, Mr. Fry was known for his philanthropy and singular modesty of life to everyone in Bristol, and to institutional workers in particular as the president and treasurer of Bristol General Hospital. It need hardly be added that he was a very generous contributor to its funds, and when, at the beginning of last autumn, the extension of the General Hospital was announced, Mr. Fry described the proposals at the half-yearly meeting, and declared that the majority of the committee was in favour of proceeding with the alterations at once notwithstanding the unknown possibilities of the Insurance Act. The extension scheme outlined by him at this time included accommodation for the resident staff, a new dental department, to take the place of the building where that work was done at a distance from the hospital, and certain additions to the wards and to the administrative department. The cost of the scheme was estimated at £45,000. It would be difficult, if not indeed impossible, to enumerate the benefactions which he made, but among the more prominent of those which came to light was his gift of £10,000 to the Convalescent Home which was founded at Bristol in memory of Queen Victoria's Diamond Jubilee. He was made a freeman of the city in 1909, and his loss will be widely felt.

### "THE IDEAL CHAIRMAN."

We have received the following appreciation of Mr. J. S. Fry from one who worked with him for many years:—"Mr. Fry, who was in his eighty-seventh year, had for a considerable time been in failing health. He was born on August 6, 1826, in Union Street Bristol, in a house which has now become part of the huge pile of 'Fry's' buildings. He was the eldest son of Mr. Joseph Fry, whose other sons were Sir Edward Fry (who was made Lord Justice of Appeal in 1883), Mr. Albert Fry (late managing director of Bristol Wagon Works), and the Right Hon. Lewis Fry. He was all his life a remarkable illustration of the keen business man and practical Christian. His energy—even when an octogenarian—was remarkable. For very many years he arrived daily at Union Street in time to conduct the short religious service with which the day's work was begun. While a consistent member of the Society of Friends Mr. Fry sympathised with all kinds of movements which commended themselves to his judgment, and he was constantly in request to preside over gatherings of different religious bodies. Mr. Fry was the ideal hospital president—princely in philanthropy, wise in counsel, and untiring in interest. He first took his place on the committee in 1886, later becoming chairman, and was elected president in 1907. It may be safely said that no place was nearer to his heart than the hospital. Even when his health and eyesight were becoming impaired, the regularity and length of his visits to the institution were the admiration of all who knew him. He was always ready to give substantial sums of money towards any project which would further its advancement, but what was still more to be appreciated was the unsparing way in which he devoted his energies to the consideration of how best this great charitable agency should be guided. At the present time, when an enlargement of the hospital is being actively proceeded with, he will be sadly missed, but the memory of his life will be an inspiration."

### NON-PANEL SOCIETIES.

The London Medical Committee recommends that an Association of Non-Panel Doctors for the County of London should be formed, consisting of those who will not agree to serve under the Insurance Act unless certain amendments are adopted. These may be summarised as free choice of doctor; an income limit; no one section to have a permanent majority on any insurance committee; and medical benefit to be in the hands of the Medical Committee. The London Medical Committee proposes to act as the central body to the many local non-panel societies which exist in London. The Secretary of the London Medical Committee, whose address is 45 Welbeck Street, will answer any communications. This is only the latest attempt at the co-ordination of the profession, of which many have been seen of late, and those who want to know what strict co-ordination and trades unionism can do may be referred to the articles on the National Medical Guild, the first of which appeared last week. The second will be found at the end of the present issue.

### PERSONS WHO HAVE NOT CHOSEN A PANEL DOCTOR.

After considerable discussion the Hull Insurance Committee passed the following resolution which appeared on the minutes of the medical sub-committee: "That the Insurance Committee be recommended to pay the practitioners on the panel the sum of £900 on account of the moneys due to them in respect of persons who have not selected a doctor during the first quarter, and also a sum in respect of the second quarter based on the mean of the numbers on the practitioners' lists at the beginning and end of the quarter." Alderman Askew urged that the matter be deferred until the legal view on the subject had been obtained, but the doctors' arguments that their remuneration depended on the rate being applicable to good and bad cases alike made their impression, and the minutes eventually were adopted.

### HOSPITALS AND FAMILY HISTORY.

We have often remarked on the way in which the voluntary hospital system creates in many families a tradition of personal service for hospitals which sometimes continues for generations. A recent example is the selection of Mr. Ernest H. L. Wilde as solicitor of St. Bartholomew's Hospital. He succeeds his father, who has retired after thirty years of work, whose father and, we believe, grandfather all in turn have occupied this post. It is probably more frequent in the provinces to find a family history intertwined with that of a voluntary hospital, but wherever it is found it is symbolic of the personal interest which voluntary work alone can create and foster.

### TRIBUTE TO AN INFIRMARY WOMAN DOCTOR.

Dr. Mabel H. Naylor is leaving Camberwell Infirmary after eight years' work there as assistant medical officer. The chairman of the infirmary committee, on behalf of the Guardians, expressed their regret at her leaving after so long a period of admirable work and devotion to the patients. She has either assisted or given anæsthetics at over 4,000 operations during her stay, and, it was mentioned, has become expert in all branches of her professional work. The Guardians felt that they were losing one who had never spared herself for the welfare of the patients, and by her exemplary work Dr. Naylor had endeared herself to all who came in contact with her. The medical and nursing staff of the infirmary presented her with a fruit epergne as a token of their admiration of her professional skill and regret at her leaving them. Dr. Naylor is marrying Dr. Ruxton, of Brentford.

### MEMORIALS IN THEORY AND PRACTICE.

The late Canon Barnett was one of those prominent men on whose death a memorial was sure to be proposed, but the attempt has been somewhat nipped in the bud by the highly interesting circular letter which Mrs. Barnett has issued describing his and her views about memorials generally. It is a letter from which our readers will be glad to have full extracts, because institutions have often profited by memorialising the dead, and officials have therefore a direct super-selfish interest in the whole question. Canon Barnett "did not like," we read, "any form of organised grief, nor the using of sacred sorrow to pay off charitable debts, nor to relieve workers from life-producing effort by endowments, nor to establish funds which, as time goes on, must cumber the ground and hinder growth, nor to erect big monumental statues, nor to buttress failing philanthropic societies." These negatives are fairly comprehensive. We go on, however, to learn that there are "many things which could be done by individuals or groups of friends without any organised appeal" in honour of his memory. Scholarships from elementary schools to Oxford; sympathetic University teaching in great centres of industry; the erection in the form of mosaics of copies of great pictures on the fronts of hospitals and public buildings suggesting thoughts to the passers in the streets; guides to show the ignorant and the young how to visit historic buildings and how to enjoy the wonders of the country; the provision of open spaces. These are among the suggestions thrown out to individuals and groups of friends, who from knowledge and affection for the man would only act to perpetuate the spirit of the departed. So they all make for higher methods than those Canon Barnett properly nipped in the bud.

### BETTER THAN MEMORIALS.

The fact is that various things want doing which need an impetus to set them on foot, an impetus which the death of a prominent man may well provide. Indeed, it may be asked, why should a man's usefulness stop at his death? In the case of the greatest men it begins rather than ends, and opportunities for improving the occasion are not so many that the common wish to memorialise the memory of those who have made their mark can be dispensed with. So Canon Barnett's attempt to discriminate between the more and the less useful forms of memorials should fructify. If it results in making it infinitely more difficult and, as we could hope, impossible for any group of interested people or persons with a hobby to fasten upon dead men's reputations and exploit them to get money for their particular schemes, the world will be the better for it. We hold as the result of a life's experience that the friends of great lives who survive should always combine to save the memory of their honoured dead from such contamination. It is the spirit and motive which underlay the life's work of the departed which should be maintained and spread abroad through any form of commemoration which can rightly be described as a memorial to the best of the race.

### OVERCROWDING AT NORTH EVINGTON INFIRMARY.

A serious state of overcrowding is reported at North Evington Infirmary, Leicester. While the institution is certified as having accommodation for 512, it seems that 564 are accommodated in it. The report of the special committee recommended that a separate building for children should be added, and that an inexpensive structure for consumptive men, two cottages temporarily to accommodate nurses, and an operation theatre should also be built. By the small majority of two this report, which Mr. Gibson proposed for adoption, was not accepted. The result for the moment is that nothing will be done. All over the country, partly through fear of incurring expenditure, partly through doubt as to what will emerge from the melting-pot in which Poor-Law administration is consigned at the present time, and partly, we fear, through a frank want of public spirit, Boards of Guardians are allowing overcrowding to persist until the Local Government Board interferes and brings pressure to bear to put an end to it. In any case, it can create only a bad impression to shelve a report on its first reading. The Leicester Guardians have only themselves to thank for the sharp criticisms which their inaction is exciting.

### NATIONAL POOR-LAW OFFICERS' ASSOCIATION.

At a meeting of the Suffolk Branch of the National Poor-Law Officers' Association last week, Mr. John Simonds, assistant secretary to the Association, said that the Poor-Law question would be dealt with shortly, and that the Unionist Social Reform Party were in favour of the abolition of the Guardians. This meant danger to the Poor-Law service, and the Association needed strengthening now to meet it. The bulk of the service, fifty thousand, had left to eleven thousand the fighting tasks. Mr. Simonds dwelt on the part played by the Association in forming the Poor-Law Examination Board. Thanks to it the examination scheme was now extended to masters and matrons. He mentioned the condemnation of the use of the words "without encumbrance" in advertisements, in which The Hospital, by the way, took part. He also remarked that when a Poor-Law officer's post was abolished compensation had been paid according to the Treasury scale, and this provided for certain years being added to the term served in assessing the compensation. They were trying to get this restored, as it had been abandoned two years ago. The speaker concluded by giving examples of the help given by the Association to individual members, and the President, Mr. Leonard Greenhalgh, described the meeting as the most successful yet held.

### PATIENTS' POCKET MONEY.

We are glad to supplement our recent account of the inmates' sale of work at the Royal Hospital for Incurables, Putney, by stating that the sale realised £580. This is, we understand, a fair average total, but it certainly means that much work remained unsold. This is very unfortunate, for very few patients enter the hospital until their friends, through death or other causes, are unable to help them, and the cost of providing their own clothing is often a very difficult matter. It is just here that the bazaar money comes to their rescue, for clothes, of course, have to be provided before little personal luxuries can be afforded. We have said enough to show how much the patients need the money, and for the many points of interest in the sale itself readers must be referred to our issue of June 28.

### THE COX MEMORIAL COTTAGE HOSPITAL.

Mrs. Cox, of Cardean, is giving a cottage hospital to Meigle and Alyth, Scotland, in memory of her late husband, Mr. Edward Cox. The final arrangements are not settled, but the donor believes that the normal needs of the Alyth and neighbouring parishes for non-infectious cases will be met by the building of a men's and a women's ward with two beds apiece, and two private wards. There will also be, of course, an operating theatre, and patients are to be attended by their own doctor. In addition, no subscriptions, said Mrs. Cox in announcing her gift at a meeting of Alyth Nursing Association last week, will be solicited. A small charge will be made upon those patients in the public wards who are in a position to pay, and the churches in the district will be expected to hold an annual Hospital Sunday collection. It will be interesting to see whether these means prove sufficient to maintain the institution.

### A GRACIOUS RECOGNITION.

No one who has had a wide experience of hospital life can fail to recognise the indebtedness of the medical and surgical staff to those members of the nursing staff in charge of their patients. On the other hand, no experienced administrator would deny the deep obligation of the nurses to the medical and surgical staff of all grades. In these circumstances it is fitting that there should be the sincerest good will and co-operation amongst the whole of the working staff of every hospital. Bearing these facts in mind, the gracious and appropriate action of Sir George Beatson, K.C.B., M.D., in establishing an annual nursing prize to be awarded to the best nurse in each year at the Western Infirmary, Glasgow, will be widely recognised. Sir George Beatson expresses the consciousness of his professional indebtedness to the Western Infirmary, and his wish to indicate how much every member of the medical and surgical staff is dependent on the skill and efficiency of the nurses of this institution. Personally, he has been most ably served by them, and he has, therefore, requested the managers to allow him to establish this annual nursing prize. Sir George Beatson proposes to hand to the managers whatever sum of money is considered necessary to provide the amount of interest required for its permanent establishment. In welcoming the course taken by Sir George Beatson, we may express the hope that his example will be followed by others, and that ultimately every voluntary hospital of importance will have a series of similar prizes to be awarded only in identical circumstances.

### THE REFORM OF DEATH REGISTRATION.

A question from Mr. Rupert Guinness as to when legislation will be introduced to render burial impossible without the production to the registrar of deaths of a medical certificate as to the cause of death, or a certificate based on the verdict of a Coroner's jury, is answered in a Parliamentary Paper by Mr. Burns. He states: "I cannot promise legislation on this subject, but an alteration which the Registrar-General is about to make, with my approval, in the instructions to registrars of deaths will secure that all deaths not certified by a registered medical practitioner shall be reported to the Coroner." Although legislative enactment of such a provision would be far preferable, at least this "instruction" will do some good by diminishing the number of deaths registered without any medical or Coroner's report as to their cause.

### HOSPITAL MEMORIAL TO AN IRISH SURGEON.

A memorial is to be raised to the memory of the late Sir Henry Swanzy, the ophthalmic surgeon. At an influential meeting held in the Royal College of Surgeons, Dublin, last week, to consider what form the memorial should take, the Honorary Secretary, Mr. G. B. Storey, stated that 280 persons had announced their willingness to serve on the general memorial committee. Eighty of these are Irish medical men, while approximately forty are medical men in Great Britain, and a similar number represents the profession in the Colonies and abroad. An executive committee was elected, and instructed, if possible, that the memorial should take the form of completing the Royal Victoria Eye and Ear Hospital in Dublin. This, of course, was the institution with which Sir Henry Swanzy was so intimately associated. We live in the age of memorials, and the age also commercially sharpened enough to see in this growing fashion of perpetuating the memories of the eminent or the successful an opportunity for improving the occasion by using the fashion as a stalking-horse for getting something done which is in need of assistance. Indeed, the rush of interests clamouring for the right to use an eminent dead man's name for their activities is in the case of kings and princes sometimes a positive scramble.

### THE HAMPSTEAD HOSPITAL'S MOTOR AMBULANCE.

The Hampstead General and North-West London Hospital has now received the motor ambulance which was kindly offered to the institution by the President, the Grand Duke Michael. In co-operation with the London County Council, who are contributing to the cost of maintenance and are arranging for special telephone facilities, the ambulance will be available for street accidents in North-West London. It will be the first motor ambulance, we understand, for street accidents in the Metropolitan area outside the City of London.

### GIFTS TO THE CAMBRIDGE RESEARCH HOSPITAL.

We are glad to announce that the treasurers of the Cambridge Research Hospital for Special Diseases have received two important donations. The first comes from Mr. G. P. Bidder, of Trinity College, who has given a sum of £300 for the purchase of an additional acre of land. The second is a promise of a similar sum from Mr. J. H. Wills, of Chiddingstone Castle, Kent, for the payment of current expenses. The hospital, which was opened a year ago, was fully described in an illustrated article which appeared in our columns on June 1, 1912. The investigation of rheumatoid arthritis and allied diseases, we may recall, is the object of the institution, and the patients who are accepted realise that they are to provide clinical material for the staff. The hospital is thus unique in its aims and methods, and it is encouraging to record the above benefactions in its support. The sum of £8,000 was asked for to endow the hospital, to which Mr. Otto Beit contributed £500 in July last.

### TUBERCULOSIS SCHEME AT NEWCASTLE.

The Newcastle City Council has adopted the report of the sanitary committee with reference to a combined scheme for the treatment of tuberculosis in the city in association with the National Health Committee. The sanitary committee propose to build a hospital with sixty-nine beds for advanced cases, adjoining the Walker Gate Hospital. The capital cost has been estimated at £9,000, towards which a grant of £5,400 is expected from the Exchequer. The annual charge on the Corporation was put down as £2,000, the balance, an equal sum, being receivable from the Exchequer. Sir Henry Newton moved the adoption of the report, urging that the scheme if adopted would give to patients in Newcastle an opportunity of recovery such as they had not had before. In seconding, Mr. Stableforth stated that since 1908 the Council had paid £2,000 for the maintenance of consumptives at Barrasford Sanatorium, and last year they agreed to subscribe £1,500 to the maintenance of a dispensary. He argued, therefore, that this scheme would cost them only £500 more. After further discussion the report was adopted unanimously.

### SMALLPOX IN SYDNEY.

The mild outbreak of smallpox which has been announced in Sydney, Australia, has led to the issue of quarantine regulations which came into force early this week. While prohibiting the passage beyond the State of unvaccinated residents of Sydney, no attempt is made, says the *Times* correspondent, to interfere with free transit within the State or to prohibit the free passage across the borders of residents outside the Sydney area. All the local Federal politicians, adds this writer, escaped unvaccinated. The State health authorities consider that a general quarantine of Sydney is unnecessary, and regard the isolation of patients and of possible contacts as a sufficient precaution. One immediate result, which may be dwelt on without impropriety, as the cases appear to be exceedingly mild, is the revival of the vaccination question. The chief result of the outbreak is expected to be the passing of an Act making infant vaccination compulsory.

### THE SCARCITY OF RESIDENTS.

All over the country in the Poor-Law infirmaries no less than in the voluntary hospitals complaints are being made as to the difficulty experienced in obtaining resident medical officers. Guardians raise the salaries offered in their advertisements, hospital managers feel prepared to accept almost any qualified man, and the pessimist shakes his head and bewails the falling off in membership of a great profession. There is no doubt an element of truth in the latter point of view, and some, though not all, of the medical training schools have to lament a serious falling off in the number of students applying for admission. The fluctuations in the membership of the medical schools attached to the London hospitals, however, are much influenced by fashion, and Oxford and Cambridge men for several years in succession show a preference now for one school, and now for another. Luckily, the popularity of the respective schools averages out in the long run to practical equality. Another factor in the scarcity of residents is, of course, the number of new professions now to be included under the general name of medicine. The school medical service in all its branches, the tuberculosis posts now open to medical men everywhere, are but two examples of the varied opportunities now offered to the qualified practitioner. In the face of this increasing competition hospitals have to offer a higher standard of experience than once was the case if their resident posts are to be filled by the right men. Here, as elsewhere, we are afraid that there is no panacea; and yet, in spite of the scarcity of residents, doctors still complain of the competition that exists in private practice.

### THE HOSPITAL TREATMENT OF SCHOOL CHILDREN.

The present arrangements for medical and dental treatment of elementary school children during the year 1913-14 between the London County Council and the authorities of twelve hospitals, twenty-one school treatment centres, and the St. Mary's, Ringwood, School will expire at the end of the present month. During the coming year it is proposed that provision shall be made for the treatment of 83,998 cases, or a net increase of 7,900. Experience has shown that the number of throat defects is now less than in previous years, and it is proposed to reduce the numbers of such cases provided for in existing agreements. As far as the hospitals are concerned the existing agreements with the governing bodies of the Charing Cross, Metropolitan Ear, Nose, and Throat, Metropolitan, Miller, Poplar, Queen's, Royal Eye, Royal London Ophthalmic, and St. Mary's, Paddington, Hospitals are to be renewed upon the present conditions. At the Belgrave Hospital for Children the number of ear, nose, and throat cases is to be reduced from 1,00C to 500 a year; at the London Hospital provision is to be made for the dental treatment of 1,540 cases annually. It is not proposed to renew the agreement with the governing body of the Woolwich and Plumstead Cottage Hospital, but a school treatment centre is to be established in a more central position in Woolwich. The total cost involved in the proposals is £23,275.

### REORGANISATION AT GUY'S HOSPITAL.

A very extensive rearrangement of the junior non-resident appointments at Guy's Hospital has been effected; and the working of several innovations will be watched with some interest by the authorities of hospitals with which large schools are in connection. The number of medical registrars has been increased to four, each receiving the same remuneration as hitherto; but each of them will also be denominated assistant medical radiographer. They work in pairs, one pair taking six months in the wards, while the other pair act for alternate weeks as radiographers and deputy post-mortem room demonstrators respectively. Each medical registrar, during his week as radiographer, attends in the *x*-ray department every day, morning and afternoon, including Saturday, and will see surgical cases in addition to medical. He will be in charge of the department in the absence of the senior medical radiographer, and will be expected to demonstrate to students. Furthermore, a dental radiographer will be appointed, who will be provided with a separate apparatus. It will be seen that *x*-rays are receiving due honour at Guy's, and that future physicians there will have had a very comprehensive training in the applications of this specialty, as well as in clinical medicine and in pathology. Two more new appointments have also been created: those of Chemical Pathologist and Morbid Histologist respectively. The former of these is an old office under a new name with increased responsibilities and emoluments; the latter is a new post entirely. It is quite clear that the authorities at Guy's are fully alive to the trend of modern work and research, and keen to mould their scheme so as to put it to the best advantage of the patients and of the school.

### YOUNG RESIDENTS AND HOSPITAL ADMINISTRATION.

The appointment of Mr. Bertram Lloyd to the vacancy on the surgical staff of the Queen's Hospital, Birmingham, has deprived Charing Cross Hospital of one of its best resident medical officers, while Birmingham gains a young surgeon who may be looked to in time to replace the loss which the Queen's Hospital sustained by the death of Mr. Jordan Lloyd. There was, by the way, no degree of consanguinity between these two gentlemen. At the time of Mr. Bertram Lloyd's appointment to Charing Cross Hospital we remarked that new blood for the resident medical officer's post was likely to prove a success, a view that has been endorsed by events. In our opinion the time is now ripe for turning the post of resident medical officer into a more senior appointment than has hitherto been the case. The duties of this post entail a very considerable responsibility not only in the professional sense, but also in the sense of guarding the good fame of the hospital in the eyes of the public. To appoint recently qualified men of untried administrative ability to control house physicians and house surgeons only very slightly junior to the resident medical officer is not often likely to be successful. It is well known that at the present time hospital administration is more difficult than ever it was, and one of the first obvious things to do is to raise the salary from the inadequate £100 now offered.

### THIS WEEK'S DRUG MARKET.

Quiet conditions continue to prevail in the Drug market, which seems rather curious, as there is little doubt that the consumption of drugs has increased not inconsiderably since the Insurance Act came into operation. A possible explanation is that distributors started with good stocks, which do not yet require to be replenished. Few changes of any note have taken place in the values of drugs during the week. Cod-liver oil is unchanged; opium is in much the same position as it was a week ago—that is to say, buyers are holding off in the hope that prices will shortly be much lower. Citric acid continues to be in good demand. Balsam tolu continues scarce. The high price of cantharides has affected the preparations of this article. Cascarilla bark is scarce. Nothing further has been announced concerning the negotiations between the cinchona-bark growers and the quinine manufacturers. The value of cloves and of clove oil continues to decline slightly but steadily.

### TO OUR READERS.

Contributions are specially invited from any of our readers to these columns. They should deal with topical subjects and news. They must be authenticated for the information of the Editor only. The minimum payment if published is 5s. There is no hard-and-fast rule as to space, but notes of about twenty lines in length are preferred.

